# Aluminium incorporation in polar, semi- and non-polar AlGaN layers: a comparative study of x-ray diffraction and optical properties

**DOI:** 10.1038/s41598-019-52067-y

**Published:** 2019-11-01

**Authors:** Duc V. Dinh, Nan Hu, Yoshio Honda, Hiroshi Amano, Markus Pristovsek

**Affiliations:** 1 Institute of Materials and Systems for Sustainability, Nagoya University, Nagoya, 464-8601 Japan; 2 School of Engineering, Nagoya University, Nagoya, 464-8603 Japan; 3 Akasaki Research Center, Nagoya University, Nagoya, 464-8603 Japan

**Keywords:** Metals and alloys, Electronic devices

## Abstract

Growth of Al_*x*_Ga_1−*x*_N layers (0 ≤ *x* ≤ 1) simultaneously on polar (0001), semipolar ($10\bar{{\rm{1}}}$3) and ($11\bar{{\rm{2}}}2$), as well as nonpolar ($10\bar{{\rm{1}}}0$) and ($11\bar{{\rm{2}}}0$) AlN templates, which were grown on planar sapphire substrates, has been investigated by metal-organic vapour phase epitaxy. By taking into account anisotropic in-plane strain of semi- and non-polar layers, their aluminium incorporation has been determined by x-ray diffraction analysis. Optical emission energy of the layers was obtained from room-temperature photoluminescence spectra, and their effective bandgap energy was estimated from room-temperature pseudo-dielectric functions. Both x-ray diffraction and optical data consistently show that aluminium incorporation is comparable on the polar, semi- and non-polar planes.

## Introduction

AlGaN-based light-emitting diodes (LEDs) operating in the deep ultraviolet (UV) spectral region (*λ* < 360 nm) have a wide range of potential applications such as water purification^1^, disinfection of medical tools^2^, as well as photo-therapy and medical diagnostics^3^. However, polar (0001) *c*-plane UV LEDs operating at below 240 nm still have very low external efficiency of less than 0.2%^4^. It is well-known that LEDs epitaxially grown along the [0001] *c*-direction have strong polarization fields across quantum-well (QW) structures^5^. These fields reduce the electron-hole wave-function overlap resulting in a reduction of the radiative recombination rate. Additionally, polar LEDs operating at *λ* < 250 nm, the light emission mode changes from transverse electric polarization (*E* ⊥ [0001]) to transverse magnetic polarization (*E* || [0001]) resulting in a reduced light extraction efficiency^6–8^.

Growth on semi- and non-polar planes results in a reduction of built-in fields^5^, which should increase the radiative recombination efficiency of QW active regions. Additionally, the optical polarization of nonpolar ($10\bar{{\rm{1}}}0$) *m*-plane Al_*x*_Ga_1−*x*_N QWs (0 ≤ *x* ≤ 1) grown on bulk AlN substrates has been found to be dominant along the *c*-direction over the entire range of composition^9^. However, despite these advantages, there are only a few reports about semi- and non-polar UV LEDs, e.g., *a*-plane AlN/non-UV-transparent-SiC LEDs operating at 210 nm^10^ and semipolar ($11\bar{{\rm{2}}}2$) AlGaN/UV-transparent-sapphire LEDs operating at 307 nm^11^. One of the reasons is lack of high-quality high-UV-transparent AlGaN and AlN templates prepared on sapphire substrates. Recently, high-temperature thermal annealing has been used to improve the material and optical properties of semi- and non-polar AlN/sapphire templates^12–16^.

In order to realize UV (visible) emitters, growth of AlGaN (InGaN) QWs with a desired Al (In) composition needs to be well-controlled. In contrast to widely compositional studies for up to twenty various InGaN surface orientations grown on GaN substrates using metal-organic vapour phase epitaxy (MOVPE)^17–22^ and ammonia molecular beam epitaxy (MBE)^23^, very limited study has been performed for semi- and non-polar AlGaN grown on AlN substrates, e.g., *m*-plane^24^, semipolar ($10\bar{{\rm{1}}}2$)^25^ and ($20\bar{{\rm{2}}}1$)^26^. This is mainly due to limit of available non-*c*-plane AlN substrates. Additionally, it should be noted that these substrates are very small and expensive, and UV-light transparency still remains a challenge^27,28^. Semi- and non-polar AlGaN layers have also been heteroepitaxially grown on sapphire substrates, e.g., *a*-plane layers on ($10\bar{{\rm{1}}}2$) *r*-plane sapphire^29^, *m*-plane^30^ and ($11\bar{{\rm{2}}}2$) layers^31,32^ on *m*-plane sapphire.

Even though semipolar ($\mathrm{10}\bar{{\rm{1}}}\bar{{\rm{3}}}$) AlN templates can be grown on *m*-plane sapphire^16^, crystal twinning has been observed for the layers that might lead to difficulties for AlGaN growth and composition determination. Recently, we have successfully produced untwinned semipolar ($10\bar{{\rm{1}}}3$) AlN templates on *m*-plane sapphire using directional sputtering^33^. Therefore, to extend compositional study of AlGaN with different surface orientations, in this paper, we report on MOVPE-growth of Al_*x*_Ga_1−*x*_N layers simultaneously on polar (0001), semipolar ($10\bar{{\rm{1}}}3$) and ($11\bar{{\rm{2}}}2$), as well as nonpolar ($10\bar{{\rm{1}}}0$) and ($11\bar{{\rm{2}}}0$) AlN/sapphire templates over the entire range of composition. Compositional study of these layers has been investigated by x-ray diffraction (XRD), room-temperature photoluminescence (RT-PL) and pseudo-dielectric functions (DFs) measurements.

## Results

### Determination of Al incorporation by XRD

**Figure 1:**
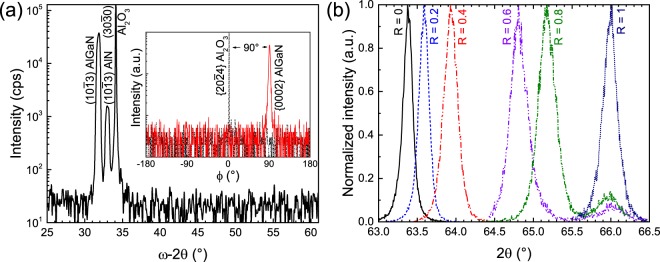
(**a**) Symmetric *ω*-2*θ* XRD scan of an AlGaN layer grown on a ($10\bar{{\rm{1}}}3$) AlN/*m*-plane sapphire template with R_AlGaN_ = 0.2. The *inset* shows azimuthal scans of the {$\mathrm{20}\bar{{\rm{2}}}4$} sapphire and {0002} Al_*x*_Ga_1−*x*_N diffraction peaks performed in skew symmetry. (**b**) 2*θ* scans of the ($10\bar{{\rm{1}}}3$) Al_*x*_Ga_1−*x*_N diffraction peaks of the layers grown on ($10\bar{{\rm{1}}}3$) AlN templates with different R_AlGaN_.

To vary the aluminium mole fraction (*x*_AlN_) of the grown AlGaN layers, different R_AlGaN_ = 2 · [TMAl]/(2 · [TMAl] + [TMGa]) gas phase ratios (0 ≤ R_AlGaN_ ≤ 1) were employed while keeping NH_3_ flow rate constantly. Figure 1(a) shows a symmetric *ω*-2*θ* XRD scan of an AlGaN layer grown on a ($10\bar{{\rm{1}}}3$) AlN/sapphire template with R_AlGaN_ = 0.2. Besides the ($\mathrm{30}\bar{{\rm{3}}}0$) diffraction peak of sapphire at 34.1°, there are only the ($10\bar{{\rm{1}}}3$) AlGaN and AlN peaks, indicating that this layer is indeed single phase.

To investigate crystal twinning and the epitaxial in-plane relationship of the grown ($10\bar{{\rm{1}}}3$) Al_*x*_Ga_1−*x*_N layers and *m*-plane sapphire, XRD off-axis *ϕ*-scans were measured. The skew-symmetric {$20\bar{{\rm{2}}}4$} sapphire diffraction peak of *m*-plane sapphire substrate was measured with a tilt angle of: 32.4°, which indicates [0001]_*sapphire*_. To indicate [0001]_*AlGaN*/*AlN*_ of the ($10\bar{{\rm{1}}}3$) layers, the skew-symmetric {0002} AlGaN peak was measured with a tilt angle of: 31.6°. The *inset* of Fig. 1(a) shows *ϕ*-scans of the {$20\bar{{\rm{2}}}4$} sapphire and {0002} AlGaN diffraction peaks of a ($10\bar{{\rm{1}}}3$) layer. Only one peak of {0002} AlGaN is found, which tilts exactly 90° with respect to the {$20\bar{{\rm{2}}}4$} sapphire peak, indicating that this layer is untwinned. The relationship is found to be [$\mathrm{30}\bar{{\rm{3}}}\bar{{\rm{2}}}$]_*AlGaN*/*AlN*_ || [$11\bar{{\rm{2}}}0$]_*sapphire*_ and [$11\bar{{\rm{2}}}0$]_*AlGaN*/*AlN*_ || [0001]_*sapphire*_.

Figure 1(b) shows 2*θ* scans of the ($10\bar{{\rm{1}}}3$) AlGaN layers grown with different R_AlGaN_. Various diffraction peak positions indicate different *x*_AlN_ of these layers. A similar result has been found for the other layers with different surface orientations.

Semi- and non-polar AlGaN layers hetero-epitaxially grown on sapphire substrates generally have triclinic and orthorhombic distortions of their wurtzite unit cells, respectively. Anisotropy in the lattice and thermal expansion mismatches along two in-plane directions results in anisotropic in-plane strain causing these distortions. This makes lattice parameter measurements, and thus *x*_AlN_ determination, difficult. By taking into account these distortions, XRD methods have been developed to determine *x*_AlN_ of nonpolar^34^ and semipolar AlGaN layers^35^.

**Figure 2:**
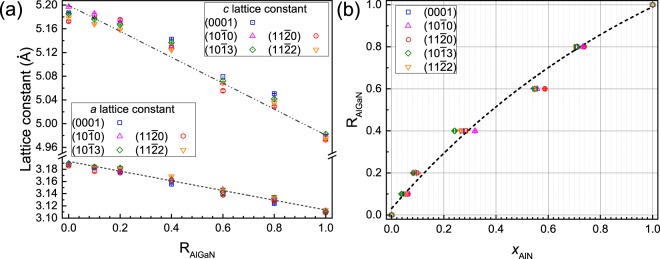
(**a**) Measured lattice constants of the differently oriented Al_*x*_Ga_1−*x*_N layers grown with different R_AlGaN_. (**b**) Calculated *x*_AlN_ from XRD data of the differently oriented Al_*x*_Ga_1−*x*_N layers plotted as a function of R_AlGaN_. Error bars in (**a**) and (**b**) are standard errors estimated from the calculations.

For the differently oriented AlGaN layers studied here, their *a* and *c* lattice constants have been calculated by measuring different symmetric, skew-symmetric and asymmetric 2*θ* diffraction peaks, as shown in Table 1. An example of lattice measurements for *m*-plane AlGaN can be seen in ref. 30. Figure 2(a) shows the measured lattice constants of the layers as a function of R_AlGaN_. The lattice constants of all the layers show a linear behaviour with R_AlGaN_. Additionally, all layers in this study have an expected ratio of *a* to *c* lattice constant with a corresponding composition, indicating that they are fully relaxed.

**Table 1:** 2*θ* XRD peaks used to measure lattice constants of the AlGaN layers with different surface orientations.

Surface orientations	Diffraction peaks
(0001)	(0002), ($\mathrm{10}\bar{{\rm{1}}}5$), ($\mathrm{20}\bar{{\rm{2}}}5$)
($10\bar{{\rm{1}}}0$)	($10\bar{{\rm{1}}}0$), ($10\bar{{\rm{1}}}1$), ($11\bar{{\rm{2}}}0$), ($11\bar{{\rm{2}}}2$), ($12\bar{{\rm{3}}}0$), ($20\bar{{\rm{2}}}1$), ($\bar{{\rm{2}}}\mathrm{021}$), ($21\bar{{\rm{3}}}1$), ($\mathrm{21}\bar{{\rm{3}}}3$)
($11\bar{{\rm{2}}}0$)	($11\bar{{\rm{2}}}0$), ($10\bar{{\rm{1}}}0$), ($10\bar{{\rm{1}}}1$), ($10\bar{{\rm{1}}}2$), ($11\bar{{\rm{2}}}2$), ($12\bar{{\rm{3}}}0$), ($2\bar{{\rm{1}}}\bar{{\rm{1}}}0$), ($21\bar{{\rm{3}}}1$), ($\mathrm{21}\bar{{\rm{3}}}2$)
($10\bar{{\rm{1}}}3$)	($10\bar{{\rm{1}}}3$), (0002), ($10\bar{{\rm{1}}}0$), ($10\bar{{\rm{1}}}1$), ($11\bar{{\rm{2}}}0$), ($1\bar{{\rm{1}}}\mathrm{02}$), ($11\bar{{\rm{2}}}2$)
($11\bar{{\rm{2}}}2$)	($11\bar{{\rm{2}}}2$), (0002), (0004), ($10\bar{{\rm{1}}}0$), ($11\bar{{\rm{2}}}0$), ($10\bar{{\rm{1}}}3$), ($2\bar{{\rm{1}}}\bar{{\rm{1}}}\bar{{\rm{2}}}$)

Based on these measured lattice constants, *x*_AlN_ of all the layers with different surface orientations has been estimated, as shown in Fig. 2(b). At each growth condition (R_AlGaN_), *x*_AlN_ values of these layers are slightly different. For example, maximum differences (Δ*x*) of 0.02/0.08/0.03 are estimated for the layers grown with R_AlGaN_ = 0.1/0.4/0.8, respectively. Given these scattered data points, *x*_AlN_ values of all the layers can be considered to be comparable. In contrast to a linear behaviour of R_AlGaN_-*x*_AlN_ observed for *c*- and *m*-plane layers grown at 1050°C reported in ref. 30, for the samples studied here grown at 1150°C, a non-linear behaviour has been observed. This is attributed to TMAl:NH_3_ pre-reactions and gallium desorption^36,37^.

### Optical properties

#### Optical bandgap energy

**Figure 3:**
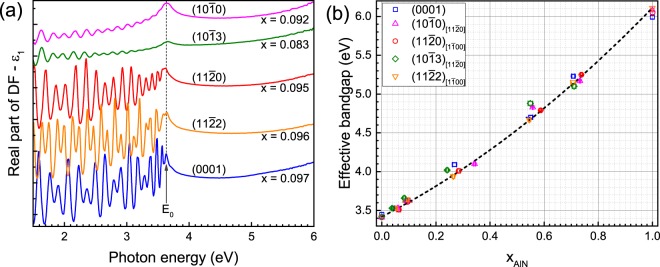
(**a**) Real part (<*ε*_1_>) of the pseudo-dielectric functions of semi- and non-polar AlGaN layers (*E* ⊥ [0001]), and of a *c*-plane co-loaded layer (*E* || [0001]) grown with R_AlGaN_ = 0.2. Effective bandgap (*E*_0_) of the band structure is indicated by an arrow. (**b**) Bandgap of these layers plotted as a function of *x*_AlN_. The dashed line is a bandgap-bowing fitting of the experimental data with a bowing parameter of 0.9 eV.

For wurtzite nitrides, the valence band maximum is split both by spin-orbit interaction and non-cubic crystal field, resulting in three valence-band states (i.e., ${\Gamma }_{7-}^{v}$, ${\Gamma }_{7+}^{v}$ and ${\Gamma }_{9}^{v}$) at the Brillouin zone centre^38^. For *m*-plane AlN^39^ and ($11\bar{{\rm{2}}}2$) AlGaN^40^, the absorption origin for *E* || [0001] indicates transitions from ${\Gamma }_{7+}^{v}$, while the one for *E* ⊥ [0001] indicates transitions mainly from ${\Gamma }_{7-}^{v}$ and/or ${\Gamma }_{9}^{v}$. Figure 3(a) exemplifies real parts (<*ε*_1_>) of the DFs measured on the differently oriented AlGaN layers grown with R_AlGaN_ = 0.2 (*x*_AlN_ ≈ 0.1). The <*ε*_1_> parts of *m*-plane and ($10\bar{{\rm{1}}}3$) layers were measured along [$11\bar{{\rm{2}}}0$]_AlGaN_, while they were measured along [$1\bar{{\rm{1}}}00$]_AlGaN_ for the *a*-plane and ($11\bar{{\rm{2}}}2$) layers. Compared to the *c*-plane, *a*-plane and ($11\bar{{\rm{2}}}2$) layers, the interference fringes of the <*ε*_1_> parts of the *m*-plane and ($10\bar{{\rm{1}}}3$) layers have weaker amplitudes because of their rougher interfaces^30^. From these <*ε*_1_> parts, the fundamental bandgap energy (${E}_{{\rm{g}}}^{{\rm{AlGaN}}}$) of the grown layers is approximately estimated from a sharp excitonic *E*_0_ peak^39–41^.

${E}_{{\rm{g}}}^{{\rm{AlGaN}}}$ of all the AlGaN layers is plotted as a function of *x*_AlN_ in Fig. 3(b). Their ${E}_{{\rm{g}}}^{{\rm{AlGaN}}}$ values are comparable over the entire range of composition. This indicates comparable *x*_AlN_ values, consistent with the values estimated by XRD (Fig. 2(b)). The dependence of ${E}_{{\rm{g}}}^{{\rm{AlGaN}}}$ on *x*_AlN_ can be described as:

$${E}_{g}^{{{\rm{Al}}}_{x}{{\rm{Ga}}}_{{\rm{1}}-x}{\rm{N}}}=x\cdot {E}_{g}^{{\rm{AIN}}}+(1-x)\cdot {E}_{g}^{{\rm{GaN}}}-b\cdot x\cdot (1-x),$$

where *b* denotes the bandgap bowing parameter. To fit the experimental data, a measured ${E}_{{\rm{g}}}^{{\rm{AlN}}}$ of 6.11 eV and a measured ${E}_{{\rm{g}}}^{{\rm{GaN}}}$ of 3.42 eV on the ($11\bar{{\rm{2}}}2$) AlN and GaN samples along [$1\bar{{\rm{1}}}\mathrm{00}$]_AlGaN_ were used, respectively. The shift of ${E}_{{\rm{g}}}^{{\rm{AlGaN}}}$ with *x*_AlN_ is well reproduced with a bowing parameter of about 0.9 eV. This value is in good agreement with values reported for *a*-plane^29^, *m*-plane^30^, ($11\bar{{\rm{2}}}2$)^40^, and *c*-plane AlGaN layers^30,41^.

#### Photoluminescence

**Figure 4:**
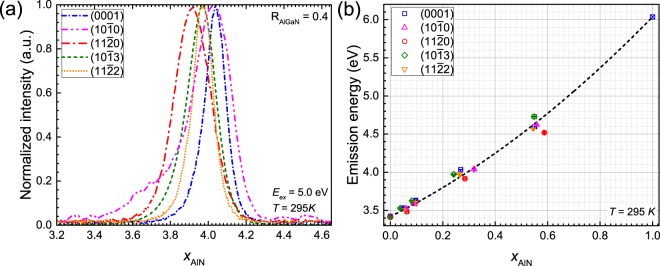
(**a**) RT-PL spectra of the differently oriented Al_*x*_Ga_1−*x*_N layers grown with R_AlGaN_ = 0.4. (**b**) PL peak energy of the layers plotted as a function of *x*_AlN_. A near band-edge energy of AlN at 6.035 eV (⊠) is taken from ref. 42. The dashed line is a bowing fitting of the experimental data with a bowing parameter of 0.9 eV.

A correlation between the bandgap energy with optical emission properties has also been investigated. Due to the excitation energy of laser (*E*_*ex*_ = 5 eV), only samples grown with R_AlGaN_ < 0.8 (i.e., *x*_AlN_ < 0.7) can be measured. Figure 4(a) exemplifies RT-PL spectra measured on the differently oriented AlGaN layers grown with R_AlGaN_ = 0.4. The near band-edge (NBE) emission energy of these samples, which was estimated from a Gaussian fit of the corresponding band, is following ($11\bar{{\rm{2}}}0$)_3.92 eV_ < ($11\bar{{\rm{2}}}2$)_3.98 eV_ = ($10\bar{{\rm{1}}}3$) < (0001) = ($10\bar{{\rm{1}}}0$)_4.04 eV_. This order is slightly different from the order shown in XRD data (Fig. 2(b)). However, the maximum NBE difference is of about 120 meV, which is equal to about a difference of 0.06 in *x*_AlN_. This composition difference is comparable with the maximum Δ*x* of 0.08 estimated from the XRD data.

The PL emission energy *vs x*_AlN_ is also well reproduced with a bowing parameter of about 0.9 eV, as shown in Fig. 4(b). For the bowing fitting, an NBE of 3.42 eV obtained from the grown GaN layers and an NBE of 6.035 eV of *c*-, *a*- and *m*-plane AlN homo-epilayers taken from ref. 42 were used. The PL data correlates very well with the optical bandgap data indicating a random alloy and almost negligible Ga clustering. This is different from the case of InGaN QWs, where a strong In clustering has often been reported, which results in a large Stokes shift between NBE and effective bandgap^43–46^.

## Discussion

Of the twenty different semi- and non-polar InGaN QWs MOVPE-grown on bulk GaN substrates investigated so far, compositional study shows different results^17–22^. For example, it has been reported that In incorporation in (0001) < ($11\bar{{\rm{2}}}2$)^19^ or (0001) ≈ ($11\bar{{\rm{2}}}2$)^18–22^, ($10\bar{{\rm{1}}}0$) < ($11\bar{{\rm{2}}}2$)^17–21^ or ($10\bar{{\rm{1}}}0$) ≈ ($11\bar{{\rm{2}}}2$)^18^, ($10\bar{{\rm{1}}}0$) < (0001)^20,21^ or ($10\bar{{\rm{1}}}0$) ≈ (0001)^18,19^, and ($11\bar{{\rm{2}}}0$) < (0001)^21,22^ or ($11\bar{{\rm{2}}}0$) ≈ (0001)^18^. This is possible that these discrepancies are due to divergence in strain relaxation in the investigated samples and/or indium clustering. For InGaN layers grown by MBE^23^, which has completely different growth kinetics from MOVPE, it has been reported that In incorporation in ($11\bar{{\rm{2}}}2$) < (0001) < ($10\bar{{\rm{1}}}0$). So far, there is only one compositional study for ($10\bar{{\rm{1}}}3$) InGaN QWs by MOVPE^21^, indicating that this plane has the lowest In-content among all the aforementioned planes.

To study In incorporation in different InGaN surface orientations, a few theoretical calculations have also been performed by taking into account surface kinetics^47,48^ or strain energy dependent surface orientations^49,50^. However, they also show contrary results, e.g., In incorporation in *m*-plane InGaN was found to be smaller^47^ or higher than *c*-plane InGaN^49,50^. Additionally, it has been theoretically^49,50^ and experimentally^19,23^ reported that different growth conditions (e.g., pressure, temperature, and V/III) might result in different In incorporations on different surface orientations.

Of the relaxed AlGaN layers with five different surface orientations studied here, their *x*_AlN_ is comparable over the entire range of composition, as consistently confirmed by XRD and optical data. The comparable *x*_AlN_ of the *m*-plane and *c*-plane layers is in good agreement with a previous report^30^, even though the growth temperature used here is 100°C higher. Given the slightly scattered data points of the *a*-plane and *c*-plane layers, their comparable *x*_AlN_ also can be considered as a consistent result with a previous report^29^, where only a slightly higher *x*_AlN_ of *c*-plane layers was found (Δ*x*_AlN_ ≤ 0.05).

For the *c*-plane and ($11\bar{{\rm{2}}}2$) AlGaN layers studied here, their comparable *x*_AlN_ is contrary to previous results reported for ($11\bar{{\rm{2}}}2$) *vs c*-plane layers, where *x*_AlN_ of ($11\bar{{\rm{2}}}2$) layers was found to be lower (Δ*x* ≤ 0.1)^32^ or higher (Δ*x* ≤ 0.2)^31^ than that of *c*-plane layers. This might be due to different growth conditions and/or calculation methods used. So far no theoretical study about composition dependent surface orientations has been done for AlGaN. In case of InGaN, most experimental data seems to indicate a higher In incorporation for orientations with almost upright metal dangling bonds. This can indicate that the bonding and incorporation of In versus In desorption are the most important step. Since the AlGaN layers studied here have a similar Al incorporation for all orientations, one may argue that the strong polarity of Al(Ga)N together with the lower total strain facilitates Ga incorporation and makes Ga desorption the less likely process. Further investigations and calculations need to be performed to clarify this.

## Conclusions

Compositional study of relaxed co-loaded AlGaN layers with polar (0001), semipolar ($10\bar{{\rm{1}}}3$) and ($11\bar{{\rm{2}}}2$), as well as nonpolar ($10\bar{{\rm{1}}}0$) and ($11\bar{{\rm{2}}}0$) surface orientations has been investigated. By taking into account the compositional effects of anisotropic in-plane strain, aluminium incorporation in semi- and non-polar layers was determined by x-ray diffraction analysis. The AlN mole fraction of all the co-loaded layers estimated by x-ray diffraction is comparable. This is consistent with their comparable optical bandgap energy and near band-edge emission energy, which were determined from room-temperature pseudo-dielectric functions and photoluminescence measurements, respectively. The dependence of the bandgap and emission energy on composition indicates a bowing parameter of 0.9 eV.

## Experimental Methods

Growth was performed in an EpiQuest 3 × 2-inch close-coupled showerhead MOVPE reactor. Ammonia (NH_3_), trimethylgallium (TMGa) and trimethylaluminium (TMAl) were used as precursors. Differently oriented AlN templates grown on sapphire substrates were used to grow AlGaN layers, including (0001) AlN (*d* ≈ 800 nm) on *c*-plane sapphire, ($11\bar{{\rm{2}}}2$) AlN (*d* ≈ 1000 nm) on *m*-plane sapphire, ($10\bar{{\rm{1}}}0$) AlN (*d* ≈ 350 nm) on *m*-plane sapphire, and ($11\bar{{\rm{2}}}0$) AlN (*d* ≈ 350 nm) on *r*-plane sapphire. The ($11\bar{{\rm{2}}}0$) AlN template was grown simultaneously with the ($10\bar{{\rm{1}}}0$) AlN template. Growth parameters of these templates are reported elsewhere^30^. To produce an Al-polar ($10\bar{{\rm{1}}}3$) AlN template, about 10-nm-thick ($10\bar{{\rm{1}}}3$) AlN layer was initially sputtered onto a 2-inch *m*-plane sapphire wafer using directional sputtering^33^. Afterwards, this wafer was loaded into the reactor chamber to grow a 300-nm-thick AlN layer at a surface temperature of 1290°C.

All the 2-inch AlN/sapphire wafers were diced into 1 × 1 cm^2^ pieces. These pieces were then co-loaded into the reactor chamber for AlGaN epitaxy. Initially, about 100-nm-thick AlN layer was grown on these templates at 1290°C at a reactor pressure of 27 hPa. Afterwards, AlGaN layers with a nominal thickness of 1.5 *μ*m were grown on these templates at 1150°C at a reactor pressure of 100 hPa. To vary *x*_AlN_, different R_AlGaN_ ratios were employed (0 ≤ R_AlGaN_ ≤ 1), while keeping NH_3_ flow rate constantly. Growth parameters of these layers are reported in ref. 30; however, the AlGaN growth temperature at 1050°C was used in that study.

The crystal orientation of the AlGaN/AlN samples was characterized using a PANalytical X’pert triple-axis high-resolution X-ray diffraction (HR-XRD) system equipped with an asymmetric four-crystal monochromator (4 × Ge220) for CuK_*α*1_ source. On-axis *ω*-2*θ* scans have been measured using an open detector without any receiving slit to distinguish between all possible orientations of the epilayers. For lattice calculations, different 2*θ* diffraction peaks of the samples were measured using an HR analyzer detector, as shown in Table 1.

For room-temperature photoluminescence (RT-PL) measurements, the samples were excited by a Krypton Fluoride (KrF) excimer laser (ExciStar XS-200) with excitation wavelength of 248 nm (*E*_*ex*_ = 5 eV) and a spot size of 50 × 500 *μ*m^2^. During PL measurements, a pulse energy of 7 mJ and a repetition rate of 200 Hz were employed, giving a power density of 5.6 kW/cm^2^. PL signals were recorded by a high-sensitivity Ocean Optics spectrometer (QE65 Pro).

The fundamental bandgap energy of the layers was estimated from real and imaginary parts of the pseudo-dielectric functions (DFs). DFs were recorded at RT using a Horiba UVISEL 2 spectroscopic ellipsometer at an incident angle of 70° and a spot size of 705 × 2030 *μ*m^2^. The photon energy was varied from 1.45 to 6.45 eV with the spectral resolution of 0.02 eV.

## References

[1] Würtele MA (2011). Application of GaN-based ultraviolet-C light emitting diodes – UV LEDs – for water disinfection. Water Res.

[2] Chen RZ, Craik SA, Bolton JR (2009). Comparison of the action spectra and relative DNA absorbance spectra of microorganisms: Information important for the determination of germicidal fluence (UV dose) in an ultraviolet disinfection of water. Water Res.

[3] Meduri NB, Vandergriff T, Rasmussen H, Jacobe H (2007). Phototherapy in the management of atopic dermatitis: a systematic review. Photodermatol. Photoimmunol. Photomed..

[4] Kneissl M (2011). Advances in group III-nitride-based deep UV light-emitting diode technology. Semicond. Sci. Technol..

[5] Takeuchi T (1997). Quantum-confined Stark effect due to piezoelectric fields in GaInN strained quantum wells. Jpn. J. Appl. Phys..

[6] Kawanishi H, Senuma M, Yamamoto M, Niikura E, Nukui T (2006). Extremely weak surface emission from (0001) c-plane AlGaN multiple quantum well structure in deep-ultraviolet spectral region. Appl. Phys. Lett..

[7] Banal RG, Funato M, Kawakami Y (2009). Optical anisotropy in [0001]-oriented Al Ga N/AlN quantum wells (*x* > 0.69). Phys. Rev. B.

[8] Northrup JE (2012). Effect of strain and barrier composition on the polarization of light emission from AlGaN/AlN quantum wells. Appl. Phys. Lett..

[9] Funato M, Matsuda K, Banal RG, Ishii R, Kawakami Y (2013). Strong optical polarization in nonpolar (1-100) Al Ga N/AlN quantum wells. Phys. Rev. B.

[10] Taniyasu Y, Kasu M (2010). Surface 210 nm light emission from an AlN p–n junction light-emitting diode enhanced by a-plane growth orientation. Appl. Phys. Lett..

[11] Balakrishnan K (2010). First demonstration of semipolar deep ultraviolet light emitting diode on m-plane sapphire with AlGaN multiple quantum wells. Jpn. J. Appl. Phys..

[12] Lin C-H, Tamaki S, Yamashita Y, Miyake H, Hiramatsu K (2016). Effects of AlN buffer layer thickness on the crystallinity and surface morphology of 10-μm-thick a-plane AlN films grown on r-plane sapphire substrates. Appl. Phys. Express.

[13] Lin C-H, Yamashita Y, Miyake H, Hiramatsu K (2017). Fabrication of high-crystallinity a-plane AlN films grown on r-plane sapphire substrates by modulating buffer-layer growth temperature and thermal annealing conditions. J. Cryst. Growth.

[14] Dinh DV, Hu N, Honda Y, Amano H, Pristovsek M (2018). High-temperature thermal annealing of nonpolar (10-10) AlN layers sputtered on (10-10) sapphire. J. Cryst. Growth.

[15] Dinh DV, Amano H, Pristovsek M (2018). MOVPE growth and high-temperature annealing of (10-10) AlN layers on (10-10) sapphire. J. Cryst. Growth.

[16] Jo M, Itokazu Y, Kuwaba S, Hirayama H (2019). Improved crystal quality of semipolar AlN by employing a thermal annealing technique with MOVPE. J. Cryst. Growth.

[17] Zhao Y (2012). Indium incorporation and emission properties of nonpolar and semipolar InGaN quantum wells. Appl. Phys. Lett..

[18] Jönen H (2012). Analysis of indium incorporation in non- and semipolar GaInN QW structures: comparing x-ray diffraction and optical properties. Semicond. Sci. Technol..

[19] Wernicke T (2012). Indium incorporation and emission wavelength of polar, nonpolar and semipolar InGaN quantum wells. Semicond. Sci. Technol..

[20] Wang Y (2015). The effect of plane orientation on indium incorporation into InGaN/GaN quantum wells fabricated by MOVPE. J. Cryst. Growth.

[21] Bhat R, Guryanov GM (2016). Experimental study of the orientation dependence of indium incorporation in GaInN. J. Cryst. Growth.

[22] Pristovsek M (2016). Structural and optical properties of (11-22) InGaN quantum wells compared to (0001) and (11-20). Semicond. Sci. Technol..

[23] Browne DA, Young EC, Lang JR, Hurni CA, Speck JS (2012). Indium and impurity incorporation in InGaN films on polar, nonpolar, and semipolar GaN orientations grown by ammonia molecular beam epitaxy. J. Vac. Sci. & Technol. A: Vacuum, Surfaces, Films.

[24] Banal RG, Taniyasu Y, Yamamoto H (2014). Deep-ultraviolet light emission properties of nonpolar m-plane AlGaN quantum wells. Appl. Phys. Lett..

[25] Ichikawa S, Iwata Y, Funato M, Nagata S, Kawakami Y (2014). High quality semipolar (1-102) AlGaN/AlN quantum wells with remarkably enhanced optical transition probabilities. Appl. Phys. Lett..

[26] Wunderer T (2017). Structural and optical characterization of AlGaN multiple quantum wells grown on semipolar (20-21) bulk AlN substrate. Appl. Phys. Lett..

[27] Collazo R (2012). On the origin of the 265 nm absorption band in AlN bulk crystals. Appl. Phys. Lett..

[28] Alden D (2018). Point-defect nature of the ultraviolet absorption band in AlN. Phys. Rev. Appl.

[29] Laskar MR (2010). MOVPE growth and characterization of a-plane AlGaN over the entire composition range. Phys. Status Solidi (RRL).

[30] Dinh DV, Amano H, Pristovsek M (2019). Nonpolar m-plane Al Ga N layers grown on m-plane sapphire by MOVPE. J. Cryst. Growth.

[31] Stellmach J (2013). Structural and optical properties of semipolar (11-22) AlGaN grown on (10-10) sapphire by metal–organic vapor phase epitaxy. J. Cryst. Growth.

[32] Dinh DV, Alam SN, Parbrook PJ (2016). Effect of V/III ratio on the growth of (11-22) AlGaN by metalorganic vapour phase epitaxy. J. Cryst. Growth.

[33] Hu N, Dinh DV, Pristovsek M, Honda Y, Amano H (2019). How to obtain metal-polar untwinned high-quality (10-13) GaN on m-plane sapphire. J. Cryst. Growth.

[34] Laskar MR (2011). Distorted wurtzite unit cells: Determination of lattice parameters of nonpolar a-plane AlGaN and estimation of solid phase Al content. J. Appl. Phys..

[35] Frentrup M, Kneissl M (2013). Determination of lattice parameters, strain state and composition in semipolar III-nitrides using high resolution X-ray diffraction. J. Appl. Phys..

[36] Keller S (1999). Metalorganic chemical vapor deposition of high mobility AlGaN/GaN heterostructures. J. Appl. Phys..

[37] Lobanova AV (2006). Effect of V/III ratio in AlN and AlGaN MOVPE. J. Cryst. Growth.

[38] Wei S-H, Zunger A (1996). Valence band splittings and band offsets of AlN, GaN, and InN. Appl. Phys. Lett..

[39] Feneberg M (2013). Anisotropic absorption and emission of bulk (1-100) AlN. Phys. Rev. B.

[40] Feneberg M (2015). Anisotropic optical properties of semipolar AlGaN layers grown on m-plane sapphire. Appl. Phys. Lett..

[41] Buchheim C (2005). Dielectric function and critical points of the band structure for AlGaN alloys. Phys. Status Solidi B.

[42] Sedhain A (2008). Photoluminescence properties of AlN homoepilayers with different orientations. Appl. Phys. Lett..

[43] Tang F (2015). Indium clustering in a-plane InGaN quantum wells as evidenced by atom probe tomography. Appl. Phys. Lett..

[44] Zhang Y (2016). Stokes shift in semi-polar (11-22) InGaN/GaN multiple quantum wells. Appl. Phys. Lett..

[45] Dinh DV, Brunner F, Weyers M, Corbett B, Parbrook PJ (2016). Exciton localization in semipolar (11-22) InGaN multiple quantum wells. J. Appl. Phys..

[46] Griffiths JT (2016). The microstructure of non-polar a-plane (11-20) InGaN quantum wells. J. Appl. Phys..

[47] Northrup JE (2009). Impact of hydrogen on indium incorporation at m-plane and c-plane In Ga N surfaces: First principles calculations. Phys. Rev. B.

[48] Northrup JE (2009). GaN and InGaN (11-22) surfaces: Group-III adlayers and indium incorporation. Appl. Phys. Lett..

[49] Durnev MV, Omelchenko AV, Yakovlev EV, Evstratov IY, Karpov SY (2010). Indium incorporation and optical transitions in InGaN bulk materials and quantum wells with arbitrary polarity. Appl. Phys. Lett..

[50] Durnev MV, Omelchenko AV, Yakovlev EV, Evstratov IY, Karpov SY (2011). Strain effects on indium incorporation and optical transitions in green-light InGaN heterostructures of different orientations. Phys. Status solidi A.

